# The effects of Fenton process on the removal of petroleum hydrocarbons from oily sludge in Shiraz oil refinery, Iran

**DOI:** 10.1186/2052-336X-12-31

**Published:** 2014-01-14

**Authors:** Mehdi Farzadkia, Mansooreh Dehghani, Maryam Moafian

**Affiliations:** 1 Department of Environmental Health Engineering, Iran University of Medical Sciences, Tehran, Iran; 2 Department of Environmental Health Engineering, School of Health, Shiraz University of Medical Sciences, Shiraz, Iran

**Keywords:** Fenton process, Total petroleum hydrocarbon, Oily sludge, Shiraz oil refinery

## Abstract

**Background:**

Due to the high concentrations of total petroleum hydrocarbons (TPH) in oily sludge and their environmental hazards, the concern regarding their effects on health and the environment has increased. The main objective of this research was focused on evaluating the feasibility of using Fenton process in removing TPH in oily sludge from Shiraz oil refinery, Southern Iran.

**Results:**

To determine optimum conditions, four different parameters were assessed at four different levels using Taguchi method. According to data, the optimum conditions were as follows: the reaction time of 1 hour, H_2_O_2_ to sample mass ratio of 15, H_2_O_2_ to Fe (II) molar ratio of 10 and pH of 5. The maximum TPH reduction rate was 36.47%. Because of the semi-solid nature of the sample and the hydroxyl radicals mainly generated in the aqueous solution, TPH reduction rate greatly improved by adding water. Ultimately, by adding 40 ml water per gram of the oily sludge under optimized conditions, the reduction rate of 73.07% was achieved.

**Conclusions:**

The results demonstrated that this method can be used as a pre-treatment method for the oily sludge. Moreover, a complementary treatment is necessary to reach the standard limit.

## Background

Crude oil contains saline, water, heavy hydrocarbons, and dirt. When crude oil is stored in refinery tanks, a dense phase is gradually formed at the bottom of the tanks called oily sludge. Considering the high concentrations of petroleum hydrocarbons in the created sludge at refineries, the California Environment Protection Agency has listed this compound as a hazardous material (K series) [1]. Petroleum hydrocarbons consist of different fractions of alkanes, alkenes, aromatic hydrocarbons and asphalts [2].

Most of these compounds cause cancer and mutations and have the potential of biological accumulation in living organisms. These compounds are resistant to biodegradation and stay in the environment for a long period of time. Disposal of the oily sludge into the environment is a threat for people as well as the environment. Therefore, the purification of the oily sludge before releasing it to the environmental is inevitably crucial [3-5].

The removal of oil pollutants are often performed by physical or chemical processes. These methods are commonly expensive with the potential of producing by- product pollutants [6]. Advanced oxidation processes are used for removing organic hydrocarbons. Fenton’s method has more advantages comparing to other methods since it is cheaper, reduced reaction time and energy consumption, non-toxic nature of the compounds, and operation and control simplicity [7].

The basis of the Fenton method is the decomposition of H_2_O_2_ and the production of hydroxyl radicals in the presence of Fe^3+^ ions as a catalyst [8-10]. Studies have shown that produced hydroxyl radicals are capable of decomposing and degrading organic contaminants such as petroleum hydrocarbons [11-16].

Lu et al. studied the remediation of petroleum-contaminated soil using Fenton method. They concluded that Fenton method increased the efficiency of the biological process [17]. In another study, petroleum-contaminated soil was treated using phosphate to increase the efficiency of the Fenton process but the reduction of more than 40% was not achieved [18]. The removal efficiency of polycyclic aromatic hydrocarbons (PAHs) was in the range of 70-98% (depending on the chemical characteristics of PAHs) using the combined biodegradation and a modified Fenton method [19].

Since Fars (in Southern part of Iran) enjoys the top rank in oil refinery in the country in recent years, there is a concern regarding the effect of petroleum hydrocarbons in oily sludge on people’s health and the environment. Therefore, the objectives of the study were to (i) evaluate the feasibility of using Fenton method in removing petroleum hydrocarbons in oily sludge obtained from Shiraz oil refinery, (ii) determine the optimum conditions using Taguchi method so that the standard limit can be achieved by further complementary treatment.

## Methods

The oily sludge sample was obtained from the bottom of one of the crude oil tanks at Shiraz oil refinery and stored at 4°C until they were used. The tank was drained after 2 years because of some repairs. The sample kept at standard conditions [20]. Data regarding the chemical and physical properties of oily sludge sample is summarized in Table 1.

**Table 1:** The chemical and physical properties of the oily sludge sample at Shiraz oil refinery

**Test**	**Test method**	**Result**
SP^1^ ( 15.56 °C)	ASTM D 4052	0.9163
Water content (%)	ASTM D 95	26
Wax content (%)	UOP 46	33.3
Drop melting point (°C)	IP 36	79
Sediment by extraction (%)	ASTM D 473	10.5
Nickel content (%)	AAS	0.01
Vanadium content (%)	AAS	<0.01
Iron content (%)	AAS	0.4
Lead content (%)	AAS	<0.01
Sodium content (%)	AAS	0.3
SiO_2_ content (%)	Gravimetric	2.2

1 specific gravity.

The standard methods (State Department of Natural Resources, Texas, US) was applied to measure TPH [20]. American Public Health Association (APHA) was used to determine the amount of humidity and iron [21]. The water content was measured by the Karl Fischer method [22].

According to Table 1, TPH concentration, iron and water content were high in the oily sludge sample while the moisture content was low. PROFEPA reported a maximum allowable TPH concentration of 2000 ml/kg in soil [23]. Due to very high concentrations of TPH in the oily sludge sample, it is very important to select a proper treatment method. In this study we used Taguchi method to determine the optimum conditions. This method is based on the effect of different parameters and the amount of response. The optimization in experimental design was performed by a limited number of tests [24-26].

The experiment was performed at a bench-scale batch reactor mode at room temperature and normal pressure. The effect of different parameters (H_2_O_2_ to sample mass ratio, H_2_O_2_ to Fe (II) molar ratio, reaction time, and pH) on the reduction rate of TPH were determined at four different levels. The retention time (1, 12, 24, and 48 hours), the molar ratio of H_2_O_2_ to Fe (II) (1, 5, and 10), the mass ratio of H_2_O_2_ to sludge (5, 10, 15, and 20) and pH (3, 5, 7, and 9) were assessed. Table 2 summarizes parameters at four different levels. Qualitek-4 software was used to design the test. The sixteen experiments were performed at two replications and the fifth factorial was used to calculate degree of freedom for determining the error [27]. Control (without Fenton’s reagent) was also used to show the effect of volatile organic content in the oily sludge.

**Table 2:** Tested parameters at four different levels

**Parameters**	**Levels**			
	**1**	**2**	**3**	**4**
H_2_O_2_ to sample mass ratio	5	10	15	20
H_2_O_2_ to Fe (II) molar ratio	0^1^	1	5	10
Reaction time (hr)	1	12	24	48
pH	3	5	7^2^	9

1 No iron added.

2 pH of oily sludge.

All chemicals were purchased from Merck (Germany). Because of the low water content of the oily sludge sample, distilled water (1 ml per 0.5 g oily sludge) was added to the sample and the pH was adjusted by sodium hydroxide (NaOH) and sulphuric acid (H_2_SO_4_). FeSO_4_.7H_2_O (99% purity) and H_2_O_2_ (30% purity) were used. After the specified reaction time, the remaining TPH was measured by Shimadzu Model gas chromatography, Flame Ionization Detector.

## Results and discussion

Table 3 shows the designed experiments and their results. As shown, experiment 12 had the highest response rate (mean 35.02%). The lowest efficiency was related to experiment 14 (1.36%).

**Table 3:** The experimental design for the reduction rate of TPH from oily sludge sample at Shiraz oil refinery using Taguchi method

**Number of experiment**	**Levels of different variables**				**TPH reduction rate (%)**	
	**H**_**2**_**O**_**2 **_**to sample mass ratio**	**H**_**2**_**O**_**2 **_**to Fe(II) molar ratio**	**Retention time**	**pH**	**1**^**st **^**repetition**	**2**^**nd **^**repetition**
1	3	3	3	3	14.1	14.26
2	3	1	3	3	8.76	8.85
3	1	4	4	3	21.41	21.53
4	2	1	2	4	4.58	46
5	4	1	4	2	11.31	11.36
6	4	4	2	1	9.41	9.51
7	2	3	4	1	15	15.05
8	4	2	3	1	34.99	35.05
9	2	2	1	3	7.66	7.74
10	4	4	1	4	23.28	23.24
11	2	4	3	2	19.87	19.87
12	3	2	4	4	35.1	34.97
13	4	3	2	3	20.89	20.99
14	1	3	3	4	1.32	1.41
15	1	1	1	1	6.2	6.3
16	1	2	2	2	22.54	22.57

### The effect of pH on the reduction rate of TPH in the oily sludge sample at Shiraz oil refinery

**Figure 1:**
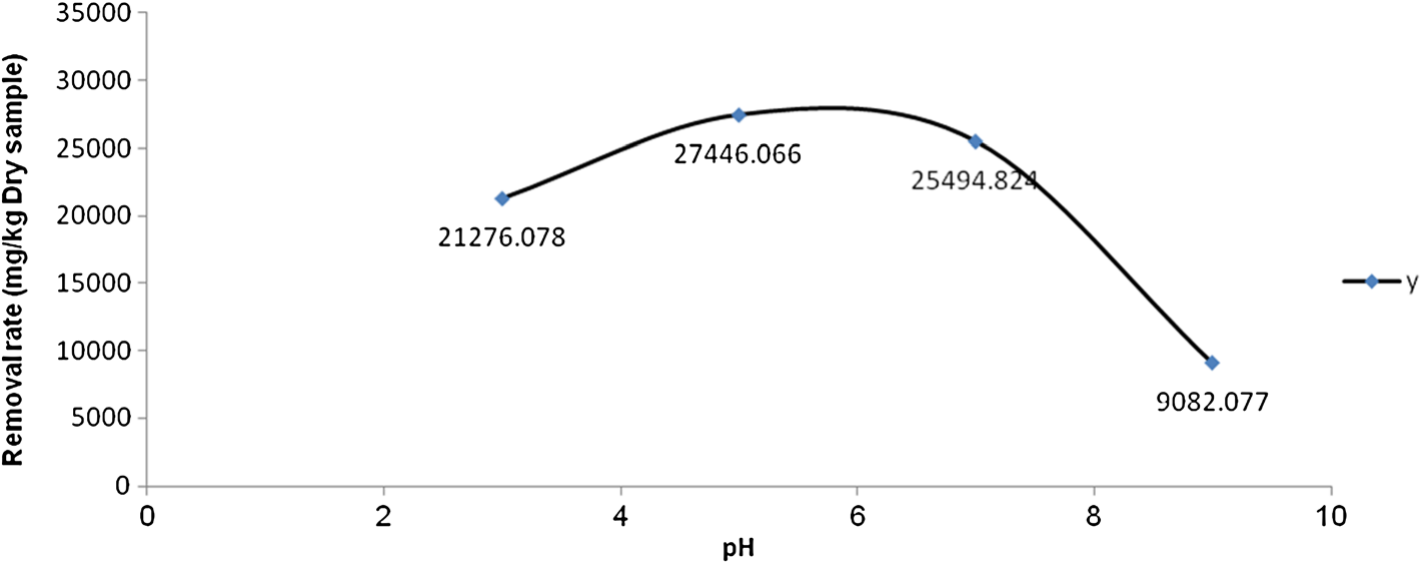
Effect of pH on the reduction rate of TPH from the oily sludge sample at Shiraz oil refinery.

The variations of pH on TPH reduction rate were shown on Figure 1. The rate of TPH reduction increased quickly when the pH increased from 3 to 5 and then reaching constant with relative slower rate up to pH equal 7 after that the reduction rate decreased when the pH increased to 9.

Due to at the production of Fe (H_2_O)^2+^ at very low pH (<2.5), the rate of TPH reduction decreased. Fe (H_2_O)^2+^ reacts with hydrogen peroxide and cause the reduction of hydroxyl radicals. Moreover, the reaction between Fe^3+^ ions and hydrogen peroxide is inhibited [28,29]. At pH < 4 the decomposition of pollutants is reduced because of the reduction of free iron ions in the solution and this can be caused by the complex formation of Fe^2+^ ions and buffer or the production of ferric hydroxide precipitate [28,29]. In alkaline conditions, the lower reduction rate is experienced as Fe^2+^ changes to Fe (OH)_3_. Fe (OH)_3_ reacts with H_2_O_2_ and inhibits the production of OH radicals. Moreover, studies have shown that the oxidative potential of OH radicals reduced as pH increased [28,29].

Many studies have shown that the optimum conditions for the Fenton process is the acidic condition. By adding the Fenton’s reagent, pH is reduced. This pH reduction has been accompanied with the production of intermediates such as carboxylic acid; therefore, the maximum reduction rate occurred at higher pH.

Kumar et al. found that the removal efficiency of chemical oxygen demand (COD) was 60% at pH = 4.27 for the coffee pulping wastewater using the Fenton process. If pH was increased to 6.4, the removal efficiency was increased to 83.9% [30]. Barbano-Arturo et al., assessed the effect of pH on the degradation of methyl tertiary butyl ether (MTBE) using the Fenton process. This study associated the pH reduction during the reaction to the production of carboxylic acids [31].

### The effect of reaction time on the reduction rate of TPH in the oily sludge sample at Shiraz oil refinery

**Figure 2:**
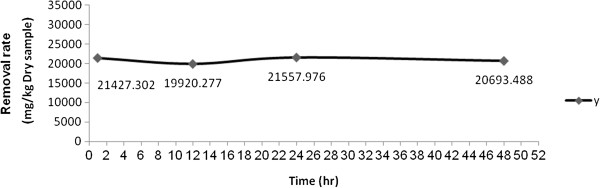
Effect of reaction time on TPH removal from the oily sludge sample at Shiraz oil refinery.

Figure 2 shows the effect of time on the reduction rate of TPH. As shown, the maximum reduction rate was achieved at the reaction time of 1 hr and then remains constant.

One of the most advantages of the Fenton process is that the reaction is very fast compared with other oxidation methods. However, the reaction time depends mostly on the type of wastewater and the amount of catalyst was used. An optimum reaction time of 90 minutes was achieved for the treatment of landfill leachate using the Fenton method. Fenton process effectively degraded high molecular weight in landfill leachate in 30 minutes [32]. The removal efficiency of 95% was achieved for TOC removal at a reaction time of 60 minutes for bamboo industry wastewater [33].

### The effect of ratio of peroxide to sample on the reduction rate of TPH in the oily sludge sample at Shiraz oil refinery

**Figure 3:**
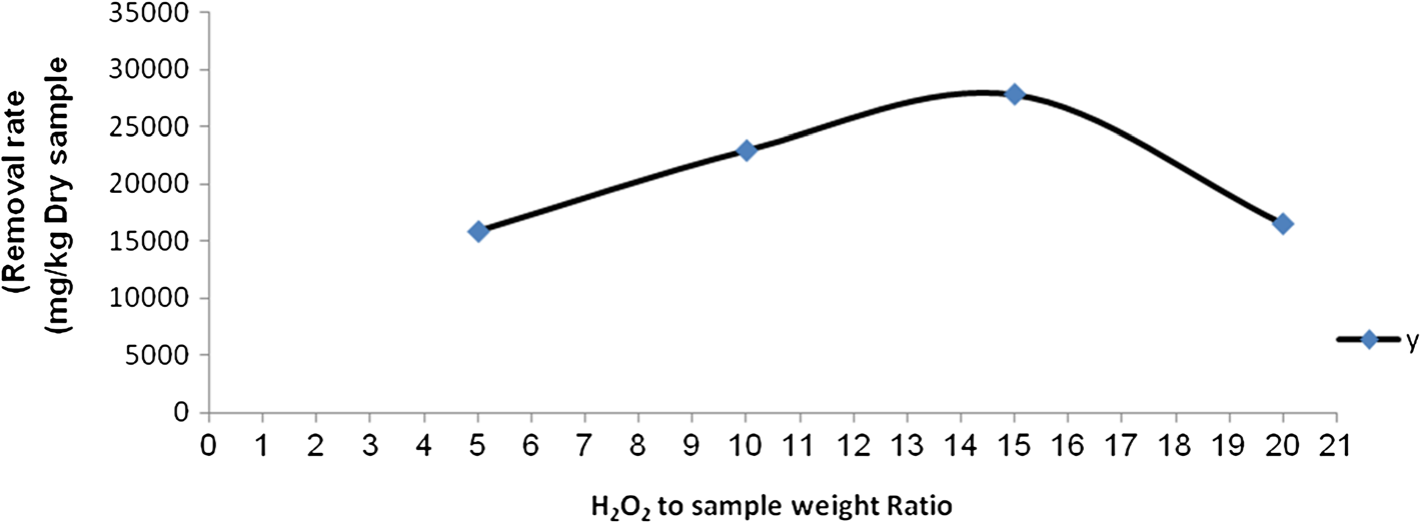
**The effect of H**_**2**_**O**_**2 **_**on the reduction rate of TPH from the oily sludge sample at Shiraz oil refinery.**

Figure 3 shows the effect of ratio of peroxide to sample on TPH reduction rate. According to Figure 3, the TPH reduction rate increased when the mass ratio increased from 5 to 15, but its reduction decreased when the mass ratio increased from 15 to 20. Therefore, peroxide concentration plays an important role on the reduction of TPH. Oily sludge contains other compounds such as heavy metals, salt, water, and also many other unknown compounds that can affect the Fenton process. Since these compounds consumed hydrogen peroxide, determining the exact amount of hydrogen peroxide is highly important. Using the higher amount of hydrogen peroxide, more than required concentration for the optimum condition, increased the COD in the effluent. Moreover, the presence of hydrogen peroxide in the effluent has toxic effect on microorganisms and decreases the feasibility of biodegradation rate. Using low concentration of H_2_O_2_ makes the Fenton process economically acceptable [32,34]. The excess amount of H_2_O_2_ acts as hydroxyl radical’s scavenger (HO_2_°) and reduced the reaction rate [35,36].

The required concentration of H_2_O_2_ for the reaction depends on the concentration and type of the pollutant. Using combined Fenton and microbial processes in a contaminated soil with benzoanthracene, the optimum amount of H_2_O_2_ was 0.3 ml per gram soil [37].

### The effect of molar ratio of H_2_O_2_ to Fe (II) on the reduction rate of TPH in the oily sludge sample at Shiraz oil refinery

**Figure 4:**
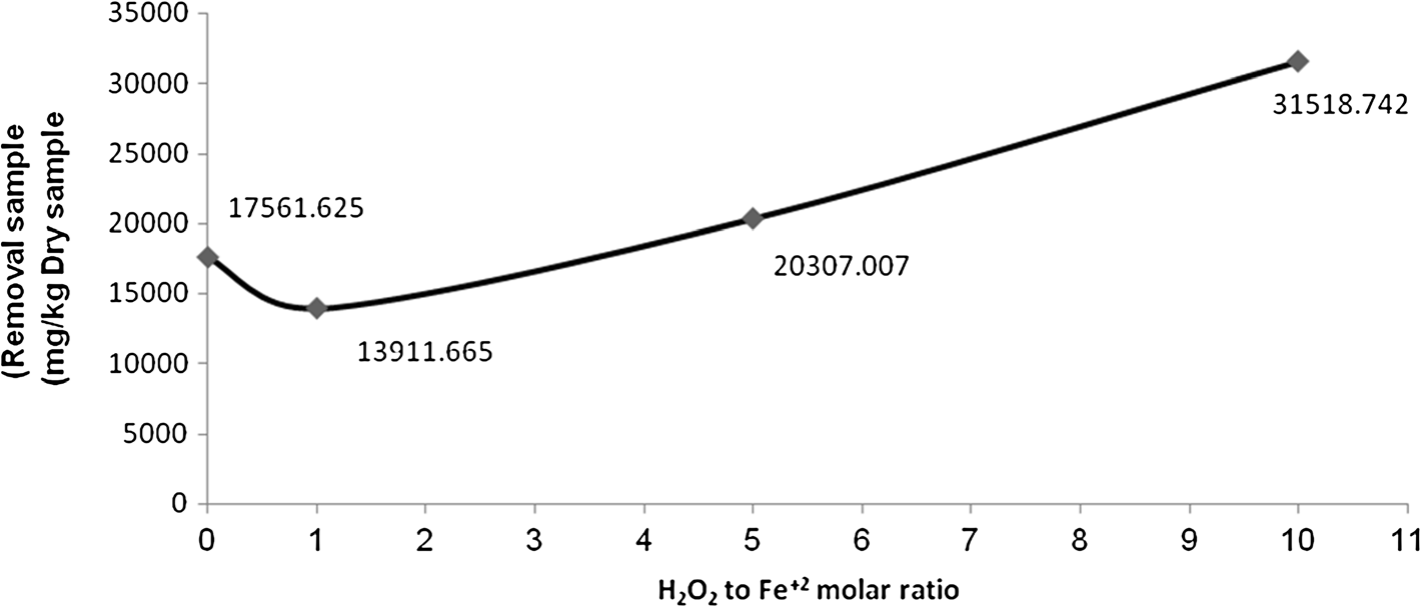
**The effect of molar ratio of H**_**2**_**O**_**2 **_**to Fe (II) on the reduction rate of TPH from the oily sludge sample at Shiraz oil refinery.**

The molar ratio of H_2_O_2_ to Fe (II) was tested at three levels of 1, 5, and 10. The oxidation of the pollutant occurred in the presence of H_2_O_2_ with or/ without the addition of iron to the sample. To examine the effect of H_2_O_2_ on the reduction rate of TPH, one of the levels was done without the addition of Fe (II). Figure 4 shows the effect of molar ratio of H_2_O_2_ to Fe (II) on the reduction rate of TPH in the oily sludge sample at Shiraz oil refinery at different levels. According to this Figure, using H_2_O_2_ without addition of Fe (II) did not have a considerable effect on the TPH reduction. In addition adding Fe (II) with the ratio of 1/1 did not increase the reduction. However, by increasing this ration to 5 and 10, the reduction rate considerably increased.

The molar ratio of H_2_O_2_ to Fe (II) is an important factor in the Fenton process. Lower ratios reduce the removal efficiency because of the reaction between excess Fe ions and hydroxyl radicals leading to the production of Fe (OH)_3_. The excess Fe would further increase the turbidity [26,38]. Because of the reaction between H_2_O_2_ and hydroxyl radicals, the higher ratios reduced the removal efficiency [38-41]. Studies have reported the different molar ratios of H_2_O_2_ to Fe (II) as the optimum ration. The amount of this ration depends on the type, concentration, and the mineral contents of the pollutant [42,43].

The Fenton process and microbial degradation was applied to remove PAHs. The optimum ration of H_2_O_2_ to Fe (II) was 10 to 1 [42]. Another study reported a similar molar ratio of H_2_O_2_ to Fe (II) 10 to 1 for removing hydrocarbons with 2–4 rings [44]. The same ratio was also obtained for the removal of aromatic hydrocarbons from soil [45].

Table 4 shows the results of analysis of variance of ANOVA. Since the four parameters were studied at four levels, the degree of freedom for comparing the response rates at 4 levels was 3. According to Table 4, the order of studied parameters regarding the effectiveness on the reaction rate was as follows: pH, H_2_O_2_ to Fe (II) molar ratio, H_2_O_2_ to sample mass ratio. The reaction time of more than one hour did not have a significant effect on the Fenton process for removing TPH from the oily sludge.

**Table 4:** ANOVA results and results analysis using the Taguchi method

**Variable**	**Degree of freedom**	**Variance (V)**	**Variance ratio (F)**	**Distribution percentage (P %)**
H_2_O_2_ to sample mass ratio	3	256330804.3	3.714	11.005
H_2_O_2_ to Fe(II) molar ratio	3	461515155.048	6.687	23.061
Reaction time	3	3696500.439	0.053	0
pH	3	543321759.458	70872	27.867
Error	19	69016758.195	-	38.067
Total	31	-	-	100.00

Using Figures and the ANOVA results, the relative optimum conditions can be obtained for the maximum reduction rate of TPH in the oily sludge sample at Shiraz oil refinery (Table 5).

**Table 5:** The optimal conditions for maximum reduction rate of TPH in the oily sludge sample at Shiraz oil refinery

**Variables**	**Optimal level**
H_2_O_2_ to sample mass ratio	15
H_2_O_2_ to Fe(II) molar ratio	10
Reaction time (hr)	1
pH	5

The optimum TPH removal conditions were determined by data analysis. Based on the analyses, the predicted percentage rate was 35.28%. According to data in this study, TPH reduction rate of 36.47% was obtained which is very close to the predicted results by Taguchi method.

### The effect of available water content on the reduction rate of TPH in the oily sludge sample at Shiraz oil refinery

Since hydroxyl radicals are formed in the aqueous phase [8], the effect of dilution was evaluated in this study. Investigation on soil samples showed that pollutant should initially be separated from the solid phase then the generated hydroxyl radicals in an aqueous phase oxidize the pollutants [46,47]. Initially, dilution was done by adding 1 ml water to 0.5 g sample. Although this amount of water was adequate for producing a solid/solution suspension, water was trapped by the sample so that the moisture content of the sample was considerably reduced. In order to increase the moisture content of the sample, different amounts of distilled water (5, 10, 20, and 30 ml distilled water) was added to reach the optimum condition for removal of TPH from oily sludge sample. Table 6 summarizes the data obtained from the reduction rate of TPH in the oily sludge sample at Shiraz oil refinery at different moisture content. According to Table 6, adding 20 ml water increased the reduction rate of TPH up to 73.03%. Increasing water content to 30 ml increased the TPH reduction rate only by 1%. Therefore, the optimum amount of added water was 20 ml to obtain the highest TPH reduction rate.

**Table 6:** The effect of moisture content on the reduction rate of TPH from the oily sludge sample at Shiraz oil refinery

**Added amounts of water**	**TPH removal percentage (1**^**st **^**repetition)**	**TPH removal percentage (2**^**nd **^**repetition)**	**Removal mean (%)**
5	40.07	40.46	40.26
10	55.56	55.43	55.49
20	72.98	73.17	73.07
30	74.12	73.89	74.00

### Evaporation of TPH

TPH reduction rate in the control reactor was 1.31%. Since all the experiments were performed at room temperature, the evaporation of TPH was quite low. Therefore, it can be concluded that Fenton’s reagent was played the main role in TPH reduction. Data obtained from this study are consistent with other studies [48,49].

### The effect of other pollutants present in the oily sludge on the reduction rate of TPH in the oily sludge sample at Shiraz oil refinery

According to the characteristics of the oily sludge sample (Table 1), it can be assumed that generated hydroxyl radicals were used for reduction of TPH and oxidation of other pollutants such as heavy metals as well.

## Conclusions

Using Taguchi method, the optimum conditions for the maximum reduction rate of TPH in oily sludge were achieved. The results demonstrated that the most effective parameters on the performance of the Fenton process were as follows: pH, the mass ratio of H_2_O_2_ to sample, the molar ratio of H_2_O_2_ to Fe (II), and the reaction time. The optimum condition of pH, the mass ratio of H_2_O_2_ to sample, and the molar ratio of H_2_O_2_ to Fe (II) were 5, 15, and 10, respectively. The reduction rate of TPH was 36.47% at optimum condition. Increasing the moisture content by diluting with water had a very effective role in enhancing the reduction rate up to 73.07%. Ultimately, the effluent TPH was 35 g/kg. This method can be a suitable pre-treatment method for treating oily sludge and adding a complementary treatment stage is necessary for reaching the desired standards.

## Competing interests

The authors declare that they have no competing interests.

## Authors’ contributions

The overall implementation of this study including design, experiments and data analysis, and manuscript preparation were the results of efforts by corresponding author. All authors have made extensive contribution into the review and finalization of this manuscript. All authors read and approved the final manuscript.
